# Ice-sheet dynamics through the Quaternary on the mid-Norwegian continental margin inferred from 3D seismic data

**DOI:** 10.1016/j.marpetgeo.2016.12.002

**Published:** 2017-02

**Authors:** A. Montelli, J.A. Dowdeswell, D. Ottesen, S.E. Johansen

**Affiliations:** a Scott Polar Research Institute, University of Cambridge, Cambridge CB2 1ER, UK; b Geological Survey of Norway, Trondheim N-7491, Norway; c Department of Petroleum Engineering and Applied Geophysics, Norwegian University of Science and Technology, Trondheim N-7031, Norway

**Keywords:** Ice stream, Fennoscandian Ice Sheet, Seismic stratigraphy, Marine geology, Palaeo-glaciology, Glacial geomorphology

## Abstract

Reconstructing the evolution of ice sheets is critical to our understanding of the global environmental system, but most detailed palaeo-glaciological reconstructions have hitherto focused on the very recent history of ice sheets. Here, we present a three-dimensional (3D) reconstruction of the changing nature of ice-sheet derived sedimentary architecture through the Quaternary Ice Age of almost 3 Ma. An extensive geophysical record documents a marine-terminating, calving Fennoscandian Ice Sheet (FIS) margin present periodically on the mid-Norwegian shelf since the beginning of the Quaternary. Spatial and temporal variability of the FIS is illustrated by the gradual development of fast-flowing ice streams and associated intensification of focused glacial erosion and sedimentation since that time. Buried subglacial landforms reveal a complex and dynamic ice sheet, with converging palaeo-ice streams and several flow-switching events that may reflect major changes in topography and basal thermal regime. Lack of major subglacial meltwater channels suggests a largely distributed drainage system beneath the marine-terminating part of the FIS. This palaeo-environmental examination of the FIS provides a useful framework for ice-sheet modelling and shows that fragmentary preservation of buried surfaces and variability of ice-sheet dynamics should be taken into account when reconstructing glacial history from spatially limited datasets.

## Highlights

• Fennoscandian Ice Sheet dynamics and associated sedimentation through the Quaternary has been reconstructed.
• Number of diagnostic buried subglacial landforms indicate complex ice-sheet evolution.
• A calving margin of the FIS was present on the Norwegian coast since the earliest Quaternary.
• Gradual development of fast-flowing ice streams started ∼1.5 Ma, with erosional intensification from ∼0.8 Ma.
• Little geomorphological evidence for subglacial drainage was found in the vast 3D seismic record.

## 1 Introduction

Deciphering the key aspects of ice-sheet development, including their past configuration, evolution of their basal thermal regime, drainage patterns and associated deposition, requires evidence from diagnostic landforms and sediments (*e*.*g*., Andreassen et al., 2004, Ottesen et al., 2005, Dowdeswell et al., 2007, Dowdeswell et al., 2016). To date, the majority of palaeo-glaciological reconstructions have focused on the Last Glacial Maximum (LGM) dynamics of ice sheets, as most of the well-preserved geological and geophysical evidence of ice-sheet activity comes from bathymetric imagery of submarine landforms preserved on the seafloor of modern polar shelves (*e*.*g*., Wellner et al., 2001, Ottesen et al., 2005). Interpretations of two-dimensional (2D) seismic records and sediment cores are valuable as they provide insights into pre-LGM histories of ice-sheet dynamics (*e*.*g*., Batchelor et al., 2013, Ó Cofaigh et al., 2013). They are, however, spatially limited since they cannot reveal key morphological parameters that are important for palaeo-glaciological reconstructions (e.g., shape, direction and elongation of glacial landforms buried up to 2 km beneath the modern seafloor). The use of three-dimensional (3D) seismic methods in high-latitude regions allows the detailed imaging of buried glacier-influenced surfaces, thus taking reconstructions of past ice sheets to a new level (*e*.*g.*, Andreassen et al., 2004, Dowdeswell et al., 2006, Laberg et al., 2010).

**Fig. 1:**
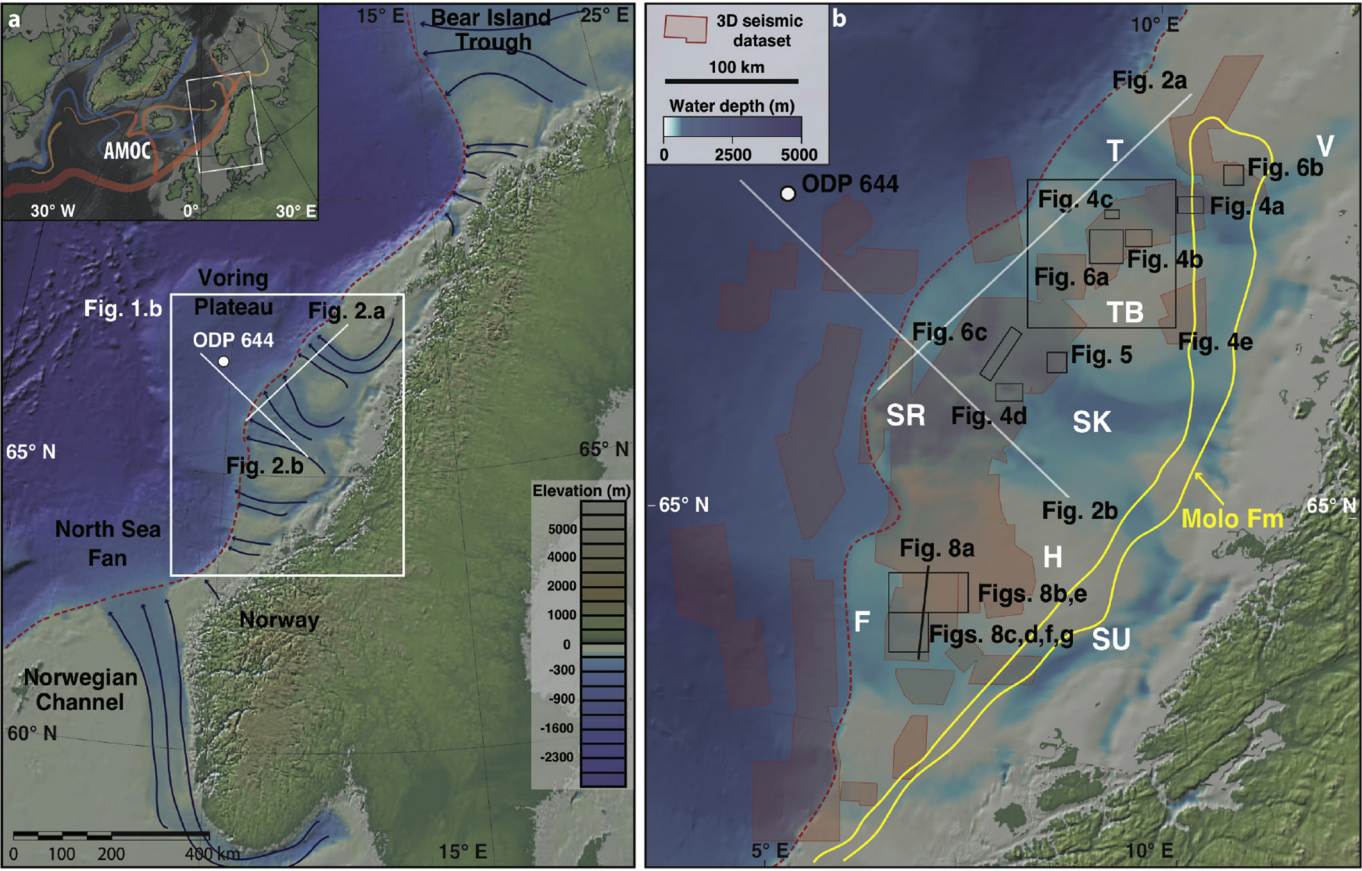
**The mid-Norwegian continental margin**. a. Dark blue lines show locations of multiple fast-flowing ice streams that drained the western margin of the Fennoscandian Ice Sheet during the Last Glacial Maximum (from Ottesen et al., 2005). Red dashed lines show the modern shelf break. Inset: large-scale ocean-surface circulation in the North Atlantic. AMOC – Atlantic Meridional Overturning Circulation. b. Study area (the mid-Norwegian continental margin between 64 and 68°N) 3D seismic data coverage of the study area (transparent red areas) and 2D seismic lines GMNR94-106 and GMNR94-302. Yellow line indicates the subcrop of the fluvial Molo Formation. T-Traenadjupet, TB-Traenabanken, SK-Sklinnadjupet, SR-Skjoldryggen, H-Haltenbanken, SU-Suladjupet, F-Frøybankhola, V-Vestfjorden. (For interpretation of the references to colour in this figure legend, the reader is referred to the web version of this article.)

In offshore Norway, exceptional coverage of 3D seismic data has resulted from decades of hydrocarbon exploration (*e*.*g*., Rise et al., 2005), providing an opportunity to elucidate, in unprecedented detail, the depositional development of the Norwegian continental margin (Fig. 1). During the Quaternary, this region was repeatedly covered by the FIS, one of the largest ice masses of the Northern Hemisphere (Clark et al., 1999, Siegert et al., 2001). The FIS has played a major role in global environmental change both directly through isostatic adjustments and ice-albedo feedbacks and indirectly through large freshwater discharges, which may have affected North Atlantic Deep Water formation and associated heat transport (*e*.*g*., Broecker et al., 1985, Clark et al., 2001). Here we use the most extensive 3D seismic datasets ever available for a high-latitude region (Fig. 1), augmented with 2D information, to reconstruct the palaeo-environment and dynamics of the marine-terminating margin of the FIS over a large part of the mid-Norwegian margin (>150,000 km^2^) during the Quaternary Ice Age of almost 3 Ma.

## 2 Background

### 2.1 Stratigraphic context

The opening of the Norwegian-Greenland Sea (ca. 55 Ma) was the major tectonic event that shaped the modern Norwegian continental margin (Hjelstuen et al., 2004). Cenozoic onshore uplift and extensive erosion led to the deposition of the Brygge (55-16 My) and Kai (16-4 My) Formations (Riis, 1996, Ottesen et al., 2009). The Molo Formation, the shallow inner-shelf equivalent of the Kai Formation, represents an extensive fluvio-deltaic unit characterized by coastal progradation during Late Miocene-Early Pliocene (Eidvin et al., 2007). Since the end of the Pliocene, repeated fluctuations of the FIS have delivered huge quantities (∼100,000 km^3^) of mainly glacier-derived sediment to the mid-Norwegian continental margin (64–68°N), producing the thick (up to 1.5 km), predominately glacigenic Naust Formation (e.g., Rise et al., 2005, Dowdeswell et al., 2010).

**Fig. 2:**
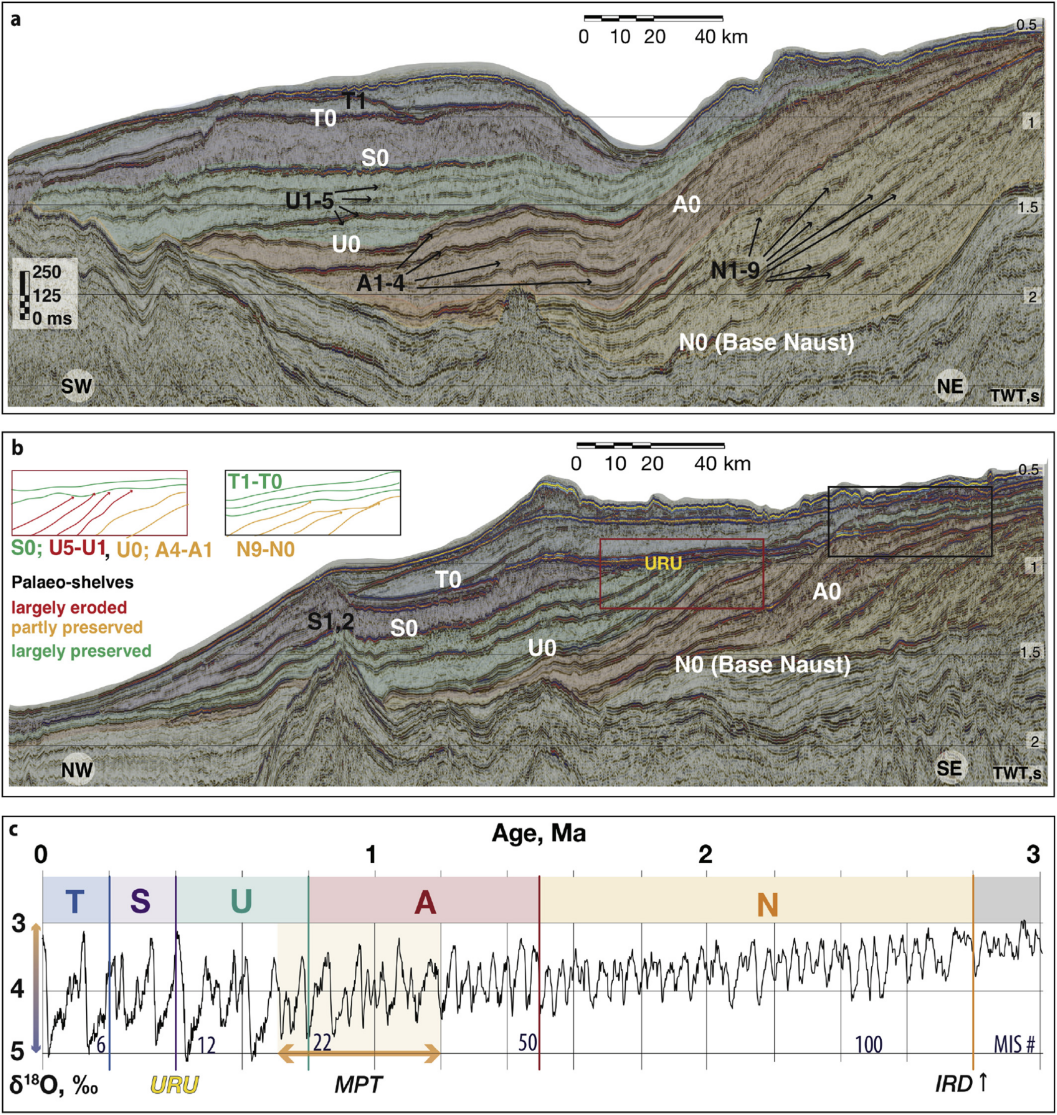
**Regional seismic stratigraphy of the Naust Formation**. a. From part of seismic profile GMNR94-302 (strike line) showing multiple coherent high-amplitude horizons (located in Fig. 1b). b. From part of a seismic profile GMNR94-106 (dip line) showing Naust sequences and degree of preservation of the buried surfaces (located in Fig. 1b). Surface N0 is the base-Naust. c. Tentative timeframe for the development of the Naust Formation (from Rise et al., 2010) juxtaposed with the δ^18^O marine-isotope curve as a proxy for global ice volume (from Lisiecki and Raymo, 2005). Annotations of major events to the left: IRD depicts ice-rafted debris increase in the early Quaternary (Jansen and Sjøholm, 1991). MPT – mid-Pleistocene Transition (1.2–0.75 Ma) (Clark et al., 2006). URU – regional erosional unconformity (Dahlgren et al., 2005;Ottesen et al., 2009).

Five major regional sequences (N, A, U, S and T) have been previously interpreted within the Naust Formation, with the ages of units S (0.4–0.2 Ma) and T (0.2–0 Ma) relatively well constrained by drill-core chronology (Dahlgren et al., 2002). Much less is known about the tentatively dated N (2.8–1.5 Ma), A (1.5–0.8 Ma) and U (0.8–0.4 Ma) sequences (Fig. 2) due to the very little sampled core material from industry wells (Rise et al., 2010, Ottesen et al., 2014). The oldest part of the Naust Formation (sequences N, A and U) has a mainly progradational character comprising low-angle wedge-shaped units (*e*.*g*., Ottesen et al., 2009). Within the younger part of the Naust Formation (sequences S and T) an upper regional unconformity (URU) has been found in many areas of the shelf (Fig. 2), marking a major change in stratal architecture (Dahlgren et al., 2005; Ottesen et al., 2009). This surface is complex, although generally coinciding with the base of the Naust S sequence, representing an erosional surface produced by the Elsterian Ice Sheet (Ottesen et al., 2009).

The mid-Norwegian continental slope is marked by several large slides that have removed glacial debris from the upper slope, affecting its sedimentary architecture (*e*.*g*., Rise et al., 2006). These slides vary in size and age, with the relatively young (ca. 4000 years ago) Traenadjupet Slide (Laberg et al., 2002), the huge Storegga Slide (ca. 8000 years ago) (Bugge et al., 1988) and the much older (ca. 250,000 years ago) Sklinnadjupet Slide located southeast of Vøring Plateau (Rise et al., 2006). The lower slope has been affected by along-slope processes and the deposition of contourites, which have often been found intercalated between individual glacigenic Naust units and infilling slide-scars along the margin (Laberg et al., 2005).

### 2.2 Fennoscandian Ice Sheet history

Sedimentation on the mid-Norwegian continental shelf has been affected by a number of advances of the FIS through the Quaternary. This sedimentary archive has been a primary source for palaeoglaciological reconstructions of the adjacent sector of the FIS (Rise et al., 2005, Dowdeswell et al., 2010). Based on the ice-rafted debris found in cores from Ocean Drilling Program boreholes located on the Vøring Plateau (Fig. 1), the onset of major glaciations in northern Europe has been inferred to have started about 2.75 Ma, coinciding with a significant increase in sediment supply to the mid-Norwegian continental margin (Jansen and Sjøholm, 1991, Hjelstuen et al., 2004, Ottesen et al., 2014). Evidence for a grounded ice sheet reaching the shelf in the early Quaternary has not been found previously on the mid-Norwegian shelf, although iceberg ploughmarks have been observed in 3D seismic data, thus indicating the presence of a calving ice front on the Norwegian coast as early as ∼2 Ma (Rise et al., 2006, Dowdeswell and Ottesen, 2013 Newton et al., 2016). Studies using long sediment cores revealed a substantial increase in ice-rafted debris (IRD) at around 1.1 Ma, suggesting a middle-Quaternary phase of major ice build-up (Jansen and Sjøholm, 1991, Sejrup et al., 1995 Rise et al., 2005, Ottesen et al., 2014). It has been suggested that the initiation of the first ice stream which began the cutting of the Norwegian Channel in the North Sea also took place at this time (Sejrup et al., 1995), but the chronological control has been questioned and it is now thought that the date of this initial ice-stream activity may be within the Brunhes magnetic period (no greater than ∼0.5 Ma) (*e*.*g*. Ottesen et al., 2014).

The development of the mid-Norwegian shelf and adjacent sector of the FIS has been relatively well studied for the last three glaciations. During the Elsterian and Saalian glaciations (∼400 ka to 135 ka), palaeo-ice streams drained the FIS through the Sklinnadjupet cross-shelf trough towards the Skjoldryggen area, switching dramatically in flow direction during the last, Weichselian glaciation (c. 20 ka) and eroding the younger Traenadjupet trough (Dowdeswell et al., 2006). During the LGM, the western margin of the ice sheet was drained by approximately 20 palaeo-ice streams that eroded the Norwegian continental shelf from Skagerrak to north of Svalbard (Fig. 1a), commonly forming cross-shelf troughs, the largest of which is 800-km long Norwegian Channel in the North Sea (Rise et al., 2005, Ottesen et al., 2005, Nygård et al., 2007, Laberg et al., 2009).

Previous reconstructions of FIS palaeo-ice streams have been focused largely on the late Quaternary and were derived from both indirect and direct evidence such as the positions of major local depocentres (*e*.*g*., Dahlgren et al., 2002; Rise et al., 2005) and the direction of streamlined glacial landforms, respectively (*e*.*g*., Dowdeswell et al., 2006, Ottesen et al., 2009). Whereas the LGM history of the FIS is relatively well-known (*e*.*g*. Hughes et al., 2016), much less is known about the growth and decay of the FIS in the early and middle Quaternary and no systematic regional-scale palaeoglaciological reconstructions for the mid-Norwegian margin have been completed over the whole Quaternary period.

## 3 Methods

### 3.1 Dataset

This study uses the Schlumberger Petrel^®^ Ready Database, investigating forty 3D seismic cubes covering ∼40,000 km^2^ of the mid-Norwegian shelf and slope, complemented by a grid of more than 150 2D reflection-seismic profiles. A composite 3D survey covers significant parts of the study area and includes multiple partly overlapping 3D cubes (Fig. 1) that have been acquired during the last two decades. The acquisitions of separate seismic surveys, both 2D and 3D, had various specifications, which generally consisted of dual sources with 25–50 m separation and 2 to 6 streamers, each of 3000–4000 m length. The depth of sources and receivers for the surveys was in the range of 5–10 m. The shot point interval was 25 or 50 m and the number of channels for each streamer was 240 or 320. The sampling rate for all surveys was 2–4 ms.

The data were processed by Schlumberger Geco and Petroleum Geo Services using standard processing software for exploration seismic-reflection data. The parameter settings of filters, velocity analysis, dip-moveout (DMO) and migration depended on multiple factors and varied for the different surveys. The best vertical resolution is 10 m assuming a dominant frequency of ∼50 Hz and sound velocity of ∼2000 m/s (Ottesen et al., 2009). The output seismic data are in two-way travel time (TWT).

### 3.2 Seismic data interpretation

Schlumberger Petrel^®^ software was used for seismic interpretation. Seismic amplitude maps and horizontal time slices were used to build structure maps of interpreted horizons and to produce isopachs of individual units. The present study uses the Naust stratigraphy from Ottesen et al. (2009) as a broad-scale regional framework for more detailed interpretations concerning individual surfaces and depocentres (Fig. 2). The identification of the most prominent, seismically coherent and continuous reflectors was performed first on the surveys of higher resolution to delineate the most reliable, uniformly widespread sequence boundaries. Each of the preserved palaeo-surfaces (horizontal resolution from 12.5 m to 50 m) was picked and extracted for further geomorphological analysis. Uncertainties in the isopach calculations are lowest (i.e., ∼5–10 ms) in the areas covered by 3D datasets and higher (up to ∼20–30 ms) where palaeo-surfaces bounding individual units have been interpolated between 2D seismic lines.

**Fig. 3:**
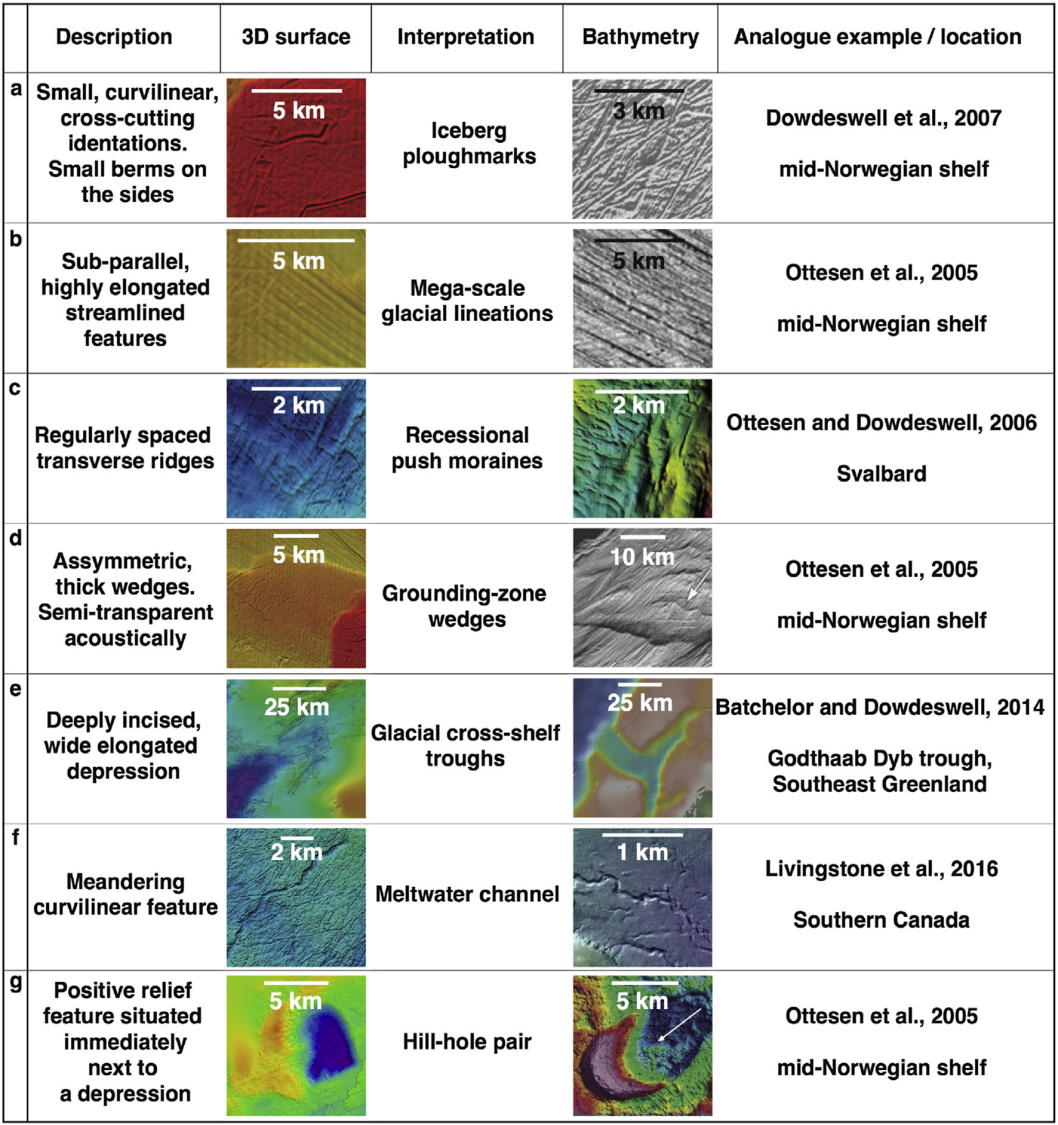
**Comparison of various buried diagnostic glacial landforms found within interpreted 3D surfaces on the mid-Norwegian margin with analogous features identified from modern high-latitude continental shelves**.

Auto-tracking methods in Petrel^®^, which extend an interpretation through the survey area, were implemented where possible. A relatively dense grid (10–50 km spacing) of 2D seismic profiles, covering the whole study area of ∼150,000 km^2^, was used to tie interpretations between individual 3D seismic cubes and to ensure quality control by cross-correlating horizons at multiple, regularly spaced intersection points. Palaeo-surfaces interpreted from high-resolution (up to 12.5 m) 3D seismic datasets along the mid-Norwegian margin contain various well-preserved landforms that range in scale and are diagnostic of past ice-sheet activity (*e*.*g*. Dowdeswell et al., 2016). Interpretation of such features requires knowledge of characteristics and morphological parameters of features observed elsewhere in glacier-influenced regions (Fig. 3).

## 4 Results I. Naust units

Within the major N, A, U, S and T sequences of the Quaternary on the mid-Norwegian margin, we identify 26 seismic units bounded by 27 regionally coherent, high-amplitude seismic reflections, most of which show a topset-truncating character on the palaeo-shelves and are therefore interpreted as erosional unconformities (Fig. 2). Here, the initial letter of the name of a palaeo-surface represents the sequence which contains that surface and numbers represent the order of interpreted surfaces within that sequence, from oldest to youngest.

15 individual wedge-shaped units, characterized by sub-parallel, low-amplitude internal reflections, were found within the progradational pattern of the oldest sequences (N and A) (Fig. 2). Basal surfaces of these individual units N0–N9 and A0–A4 show a gently dipping configuration on the continental shelf, with largely preserved outer shelves and predominantly eroded inner shelves (*e*.*g*., Dowdeswell et al., 2007) (Fig. 2b).

Sequence U was subdivided into 6 seismically semi-transparent units bounded by basal surfaces U0–U5, with the palaeo-shelves of surfaces U1–U5 being completely truncated (Ottesen et al., 2009) (Fig. 2b). Sequence S marks the regional transition in the stratal architecture of the Naust Formation from progradational clinoforms to the more flat-lying sedimentary units of Sequence T (*e*.*g*., Ottesen et al., 2009). Sequence S has a mostly irregular, chaotic internal seismic character and is disturbed by the large Sklinnadjupet Slide on the upper slope (Rise et al., 2006). Finally, two distinctive surfaces, S1 and S2, were found in the middle-slope section of this sequence. Basal surfaces T0 and T1 mark two major, aggradational, internally chaotic shelf units within Sequence T.

## 5 Results II. Buried subglacial landforms

### 5.1 Description

**Fig. 4:**
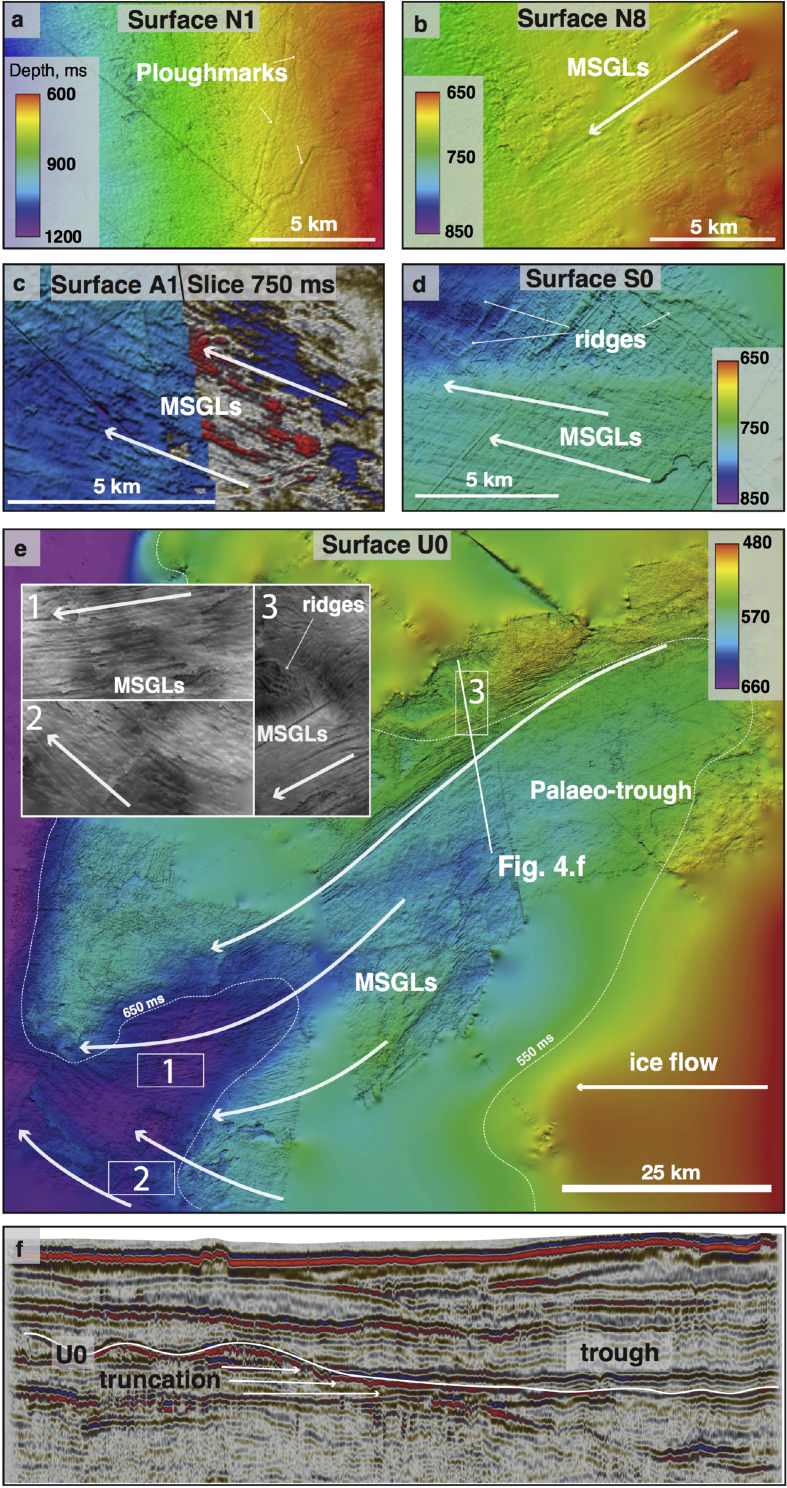
**Buried subglacial landforms preserved in the mid-Norwegian continental shelf**. a. Buried palaeo-shelf surface N1 with evidence of iceberg ploughmarks on the palaeo-shelf (3D seismic cube NLGS-95). b. Buried palaeo-shelf surface N8 with preserved MSGLs. Arrow indicates inferred direction of former fast ice flow (3D seismic cube GNNR-99). c. Left part: MSGLs found within buried palaeo-shelf surface A1 within the upper Naust Formation. Right part: horizontal seismic slice through the same surface at ∼750 ms deep showing the continuation of the same set of MSGLs (3D seismic cube GNNR-99). d. Streamlined features bordered by the transverse ridges (grey lines) found within the palaeo-shelf of surface S0, in modern Sklinnadjupet (3D seismic cube SG-9605) e. Elongate depression interpreted as a palaeo-trough within the shelf surface S0 (multiple 3D seismic cubes). f. Seismic cross-section profile across the palaeo-trough margin in panel e.

The buried shelf and slope surfaces of most Naust units are dominated by extensive, irregular, curvilinear cross-cutting depressions, typically 5–20 m deep, up to a few hundred metres wide and up to 20 km long (Fig. 3, Fig. 4, Fig. 8d, Table 1). These features show both u- and v-shaped configurations on seismic cross-sections and commonly have small berms (a few ms TWT high) on either side of the depressions.

Sub-parallel, streamlined, highly elongate ridge-groove features (Fig. 3, Fig. 4, Fig. 8) are present on multiple interpreted palaeo-shelves, although their spatial distribution within these surfaces is much more clustered and organised compared to the irregular curvilinear cross-cutting depressions described above. These features are up to 15 km long, generally a few hundred metres wide and up to 15 m high. Within sequences U, S and T sets of these sub-parallel landforms often occupy large elongate depressions (up to ∼150 m deep and tens of kilometres wide – Fig. 3e) that truncate underlying reflections on seismic cross-sections (Fig. 4e and f).

**Fig. 5:**
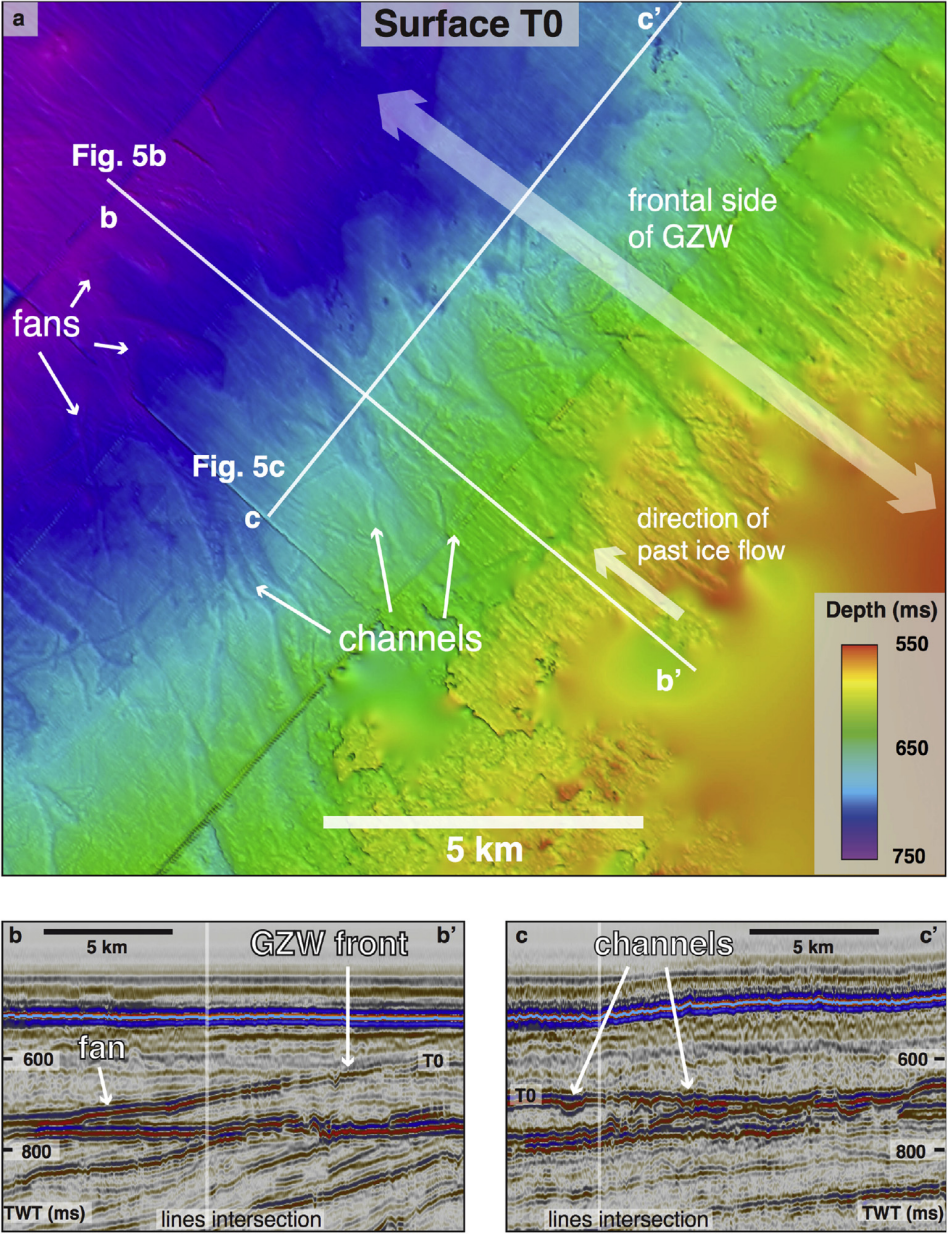
**Buried channel-fan complexes within the frontal slope of a thick (>100 m) buried wedge (3D seismic cube ST9301)**. a. Structure map of interpreted surface T0. Frontal side represents ice-distal side of the grounding-zone wedge (GZW). b. Seismic section aligned with the direction of past ice flow showing the acoustic structure of the front of the GZW. c. Seismic cross-section perpendicular to the direction of past ice flow showing channels at the distal side of the wedge.

**Fig. 6:**
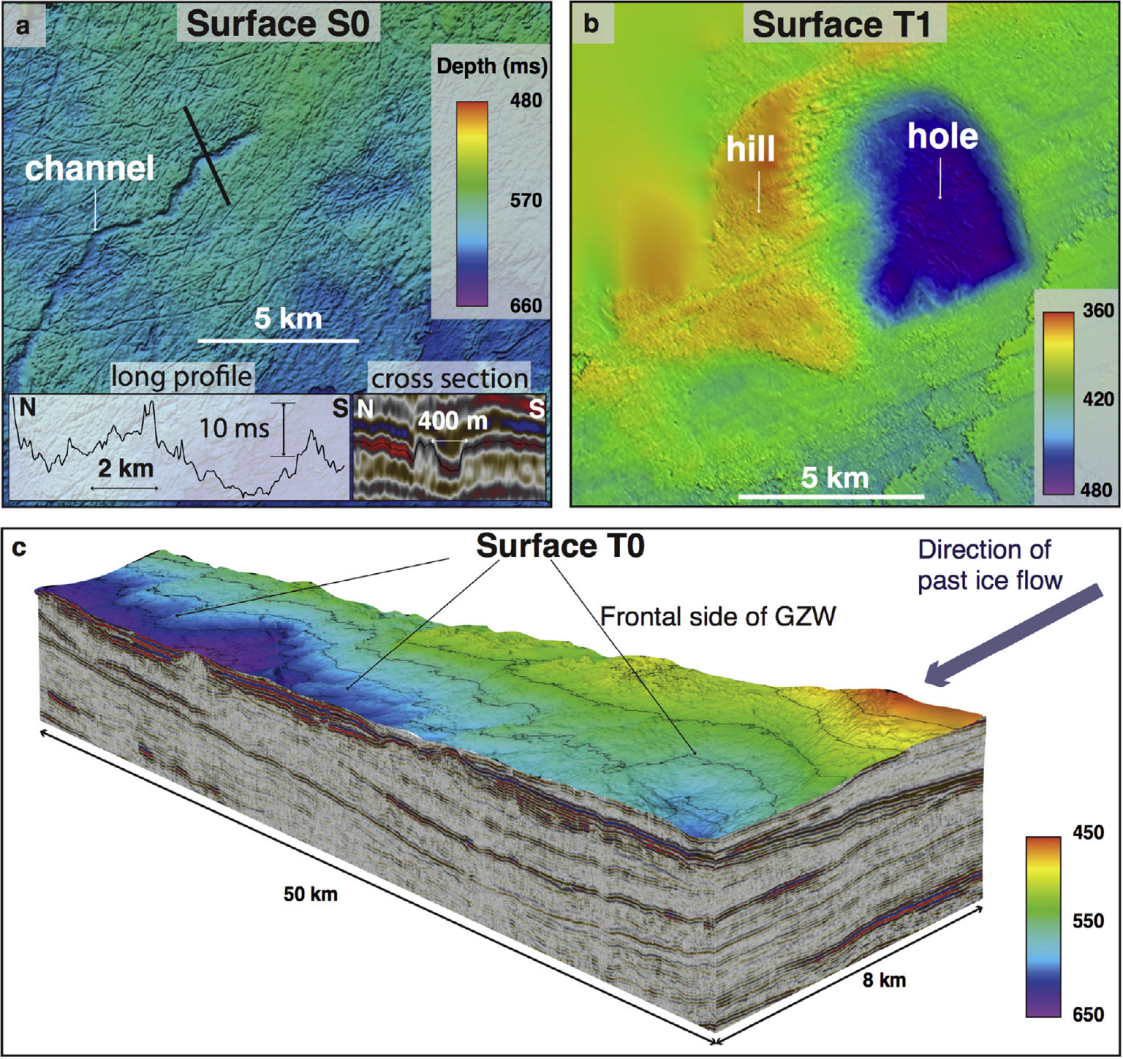
**Buried subglacial landforms preserved on the mid-Norwegian continental shelf**. a. Channel within the buried S0 surface and cross-sectional 2D line showing its seismic character (3D seismic cube GNNR-99). b. Hill-hole pair (3D seismic cube ST0107). c. 3D structure of the frontal part of grounding-zone wedge found within Unit T0 (3D seismic cube NNE-2002).

In some areas, series of overprinted, regularly spaced ridges are found on shallower banks adjacent and orientated transverse to the streamlined landforms (Fig. 3, Fig. 4d). These ridges are a few meters high and spaced several hundred meters apart. Another type of positive-relief feature, which is present mostly in upper sequences S and T, is a large (∼100 m thick), acoustically semi-transparent asymmetrical wedge (Fig. 3, Fig. 5, Fig. 6c). The topsets of these features are truncated, sometimes with channel-fan complexes on their frontal sides (Fig. 5a).

Two further landforms were identified but were seldom seen on buried Naust surfaces. The first of these unusual landforms is a ∼30 m deep depression situated immediately next to a horseshoe-shaped hill of similar magnitude, found within the Sequence T (Fig. 3, Fig. 6b). The second is a curvilinear erosional feature approximately 400 m wide and 10 km long, which is morphologically distinct from the finer-scale curvilinear cross-cutting indentations surrounding it, is present within the middle shelf part of the S0 surface (Fig. 3, Fig. 6a). This landform has a meandering character and an undulating vertical long-profile (Fig. 6a).

### 5.2 Interpretation

The irregular pattern and morphometry of buried curvilinear depressions found within multiple 3D surfaces (Fig. 3a) provide typical characteristics which identify these features as ploughmarks produced by the keels of drifting icebergs (*e*.*g*., Woodworth-Lynas et al., 1991, Dowdeswell et al., 1993). These features are similar to iceberg ploughmarks from both modern and buried shelf surfaces in other high-latitude regions (Fig. 3) (*e*.*g*., Dowdeswell and Bamber, 2007, Dowdeswell and Ottesen, 2013). The oldest evidence of iceberg ploughmarks within the Naust Formation is preserved within surface N1 immediately above the base of the Quaternary sediments (Fig. 4a).

The sub-parallel ridge-groove landforms illustrated in Fig. 3b are similar in shape, dimensions and their sub-parallel conformity to Mega-Scale Glacial Lineations (MSGLs) that have been reported widely from other glacier-influenced continental margins (*e*.*g*., Clark, 1993, Shipp et al., 1999, Canals et al., 2000, Ó Cofaigh et al., 2002, Stokes and Clark, 2003, Spagnolo et al., 2014). Such sedimentary features have also been observed forming subglacially at the base of modern Antarctic ice streams (*e*.*g*., King et al., 2009). The oldest evidence for preserved MSGLs was found within surface N8 in the upper part of Sequence N (Fig. 4b). These features are also present in most of the younger sequences (Fig. 8), where the palaeo-shelves are not eroded by subsequent ice advances, as is the case with surfaces U1–U5.

The dimensions and truncating character of large depressions (Fig. 3, Fig. 4e) suggest that they represent cross-shelf palaeo-troughs, excavated by ice streams, similar in physiography to many such troughs reported from high-Arctic margins by Batchelor and Dowdeswell (2014). MSGLs, often found to occupy the base of such troughs, provide additional evidence for palaeo-ice stream activity. Major changes in the orientation of MSGLs were found in the Haltenbanken-Frøybankhola area, where they occupy a deep and wide depression (Fig. 4e and f), which represents the oldest palaeo-trough present in the study area.

Small transverse ridges, just a few metres high, are similar in morphology to small recessional push moraines (Fig. 3c), formed annually as a result of minor winter readvances of the glacier terminus in many modern Arctic fjords (*e*.*g*., Boulton, 1986, Ottesen and Dowdeswell, 2006, Dowdeswell et al., 2007, Burton et al., 2016). By contrast, the more subdued vertical expression but large-volume dimensions of major asymmetrical wedges (Fig. 3, Fig. 6c), together with their internal acoustic character, suggest that these landforms are grounding-zone wedges (GZWs – Batchelor and Dowdeswell, 2015), formed at former ice-shelf grounding zones by the supply of poorly sorted subglacial sediments during centennial still-stands in ice-stream retreat (*e*.*g*., Dowdeswell and Fugelli, 2012). Within the Sequence T, these features are present in the large buried troughs, adding to the evidence of palaeo-ice stream activity (Fig. 6c). Subtle channels and small fans (Fig. 5) found at the ice-distal side of an acoustically semi-transparent GZW within Sequence T (∼0.2 Ma) provide evidence of meltwater activity at the grounding line. The relatively small (few metres) amplitude of these well-preserved features and their short lateral continuity (∼10–15 km) suggest possibly short-lived, episodic meltwater drainage that developed during an ice-sheet still-stand or subsequent retreat (*e*.*g*., McMullen et al., 2006, Dowdeswell et al., 2016).

A single hill-hole pair present within surface T1 is similar in morphology to both Late Quaternary terrestrial and marine analogues (Fig. 3, Fig. 6b) (*e*.*g*., Moran et al., 1980, Ottesen et al., 2005, Winsborrow et al., 2010). These glacitectonic features are thought to be formed as a result of displacement and transport of basal sediment by the overlying ice, which may be induced by the basal water freezing close to thin ice-sheet margins (Moran et al., 1980, Sættem et al., 1994).

Finally, the relatively smooth, meandering feature about 400 m wide observed on surface S0 (Fig. 3, Fig. 6a) is interpreted as a channel cut into the underlying sedimentary substrate. Its erosional character together with evidence of an irregular, undulating vertical profile indicates formation at a high water-pressure, and, therefore, it is interpreted as a subglacial meltwater channel (*e*.*g*., Larter et al., 2009), resembling those previously reported from other glacier-influenced regions (Fig. 3f) (*e*.*g*., Laberg et al., 2010, Livingstone et al., 2016). Other than this single feature, there is very limited conclusive geomorphological evidence for the presence of similar meltwater channels in 3D records of the vast mid-Norwegian shelf.

## 6 Results III. Reconstruction of Quaternary FIS evolution of the mid-Norwegian margin

**Fig. 7:**
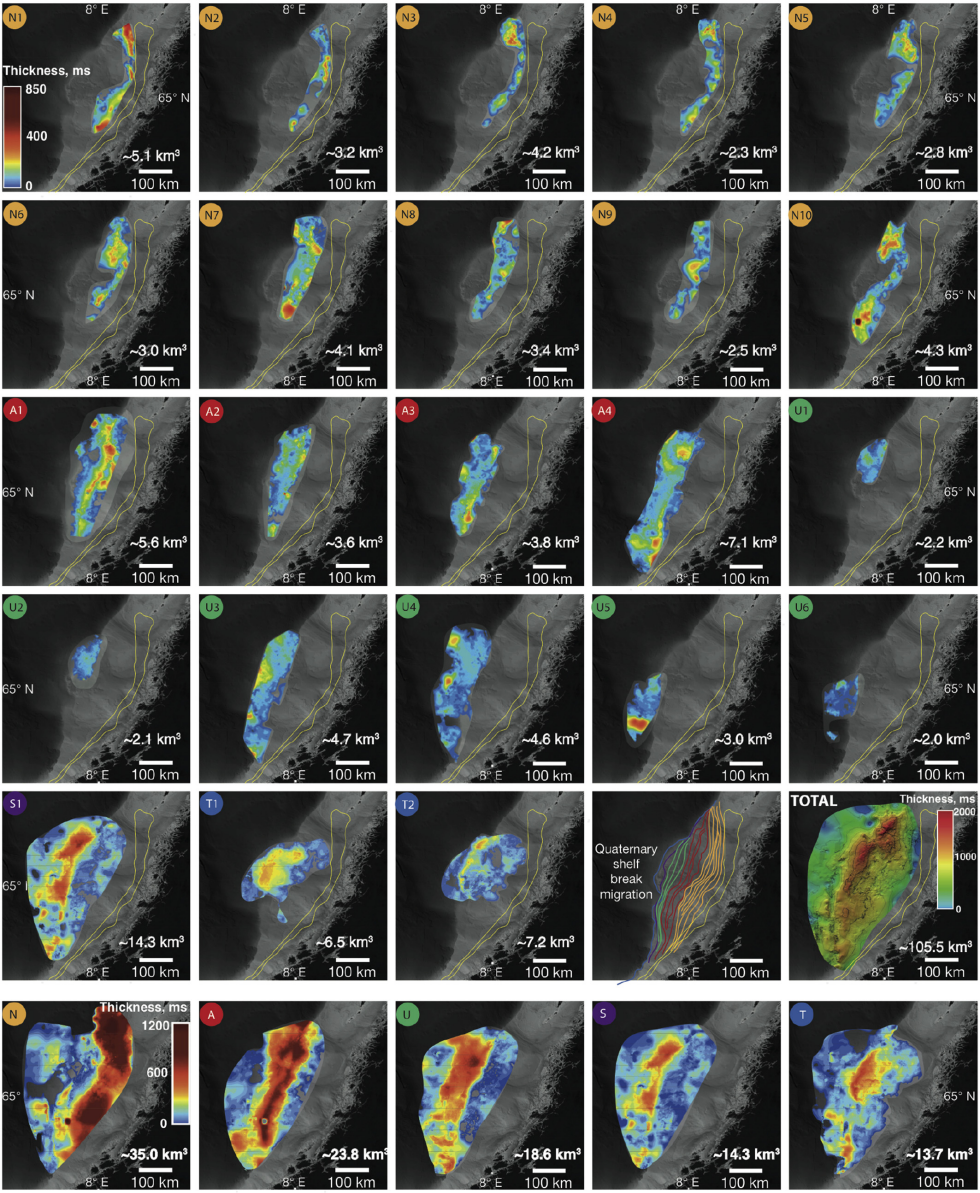
**The depositional development of the mid-Norwegian continental margin during the Quaternary**. Isopach maps of every interpreted subunit within the Naust Formation in two-way travel time (TWT), ms, show migration of major glacial depositional centres along the mid-Norwegian shelf and slope. Chronological order from left to right; each isopach is labelled according to the name of the unit (top left corner of each square). Yellow line indicates the subcrop of the fluvial Molo Formation. Fourth and fifth squares of the fifth row: Quaternary shelf break migration mapped from multiple Naust units and total thickness for the Naust Formation, respectively. Bottom row from left to right: Isopachs for N, A, U, S, T Sequences. Approximate volumes were calculated based on mean sound velocities of 2150 m/s (Dowdeswell et al., 2010). (For interpretation of the references to colour in this figure legend, the reader is referred to the web version of this article.)

The structural and thickness maps produced for individual Naust units throughout the vast mid-Norwegian margin illustrate the detailed patterns of the Quaternary evolution of this shelf-slope system (Fig. 7). At the beginning of the Quaternary, the configuration of the continental margin was asymmetrical, with a much narrower shelf and deeper adjacent accommodation space in the Traenadjupet area. Sedimentation through Sequence N, in the region of modern Traenadjupet (Fig. 7), produced southwestward-migrating depocentres as the accommodation space became gradually filled and the margin built out from one glaciation to the next. In the central and southern parts of the area, the pattern of deposition was rather poorly defined, with no stable single depocentre developing and a relatively uniform pattern of margin progradation orientated parallel to the modern coastline (Fig. 7).

During the deposition of unit A, the shelf break migrated ∼50 km seaward, with relatively dispersed depocentres developing in the Traenabanken and Frøybankhola areas. The younger Sequence U is largely eroded on the shelf, but the slope record shows the onset of slope bulging and a thick depocentre in the Sklinnadjupet area (Fig. 7, Fig. 9). The deposition of Sequences S and T marks a shift in the architecture of the margin, with an extremely prominent depocentre on the outer shelf and upper slope developing in the Skjoldryggen area.

**Fig. 8:**
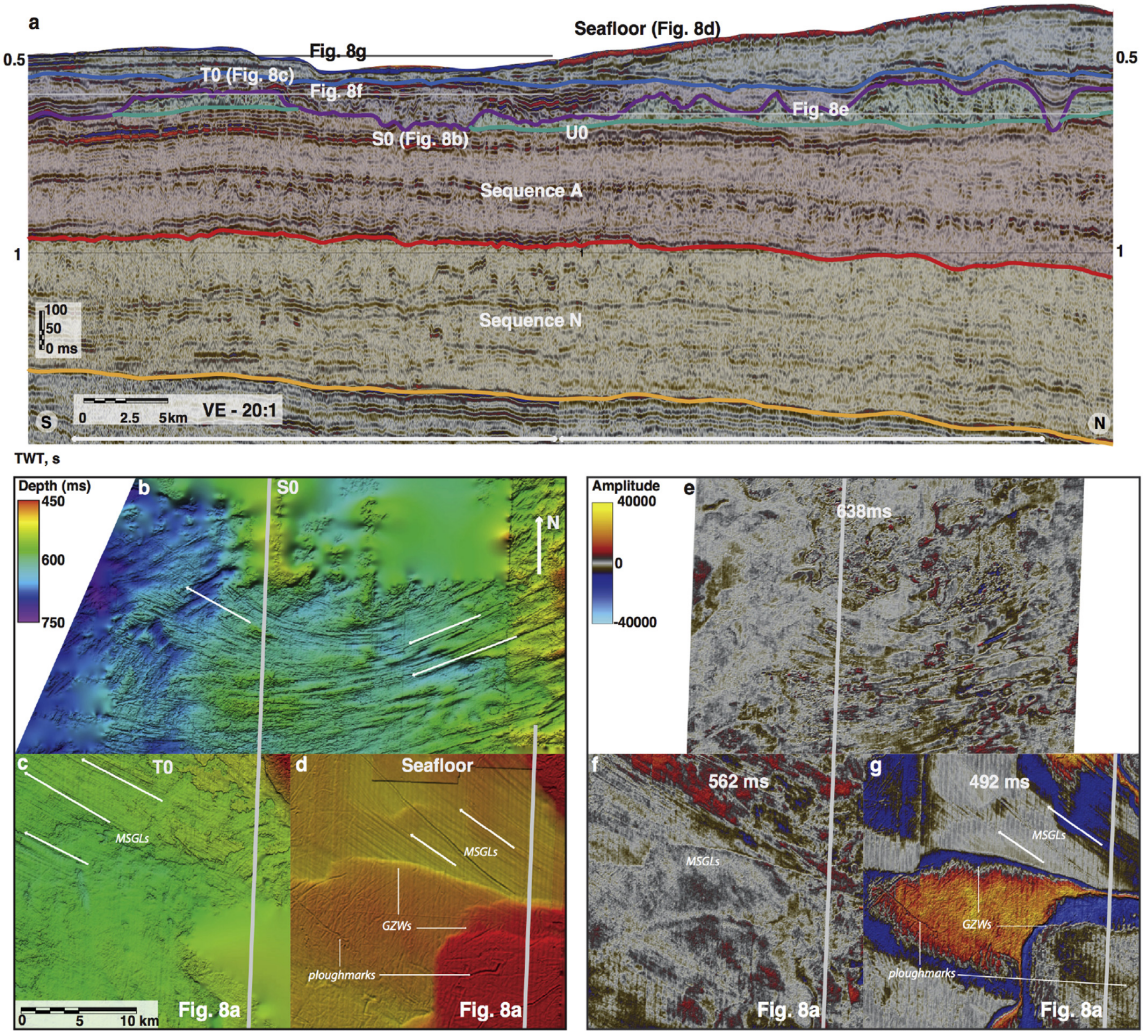
**Flow-switching observed in the Haltenbanken-Suladjupet area**. a. Composite seismic cross-section complied from two overlapping 3D cubes with location of seismic slices and key interpreted surfaces. b,c,d. Interpreted 3D surfaces S0, T0 and seafloor showing multiple subglacial landforms and changes in the direction of MSGLs. e,f.g. Seismic amplitude slices through 638, 562 and 492 ms, respectively, showing the same features in the same areas as in panels b,c, d with a different imaging method.

Migration of local depocentres may reflect changing ice-stream flow from one glaciation to another (*e*.*g*., Dowdeswell et al., 2006). We integrate our observations of depocentre development with evidence from buried landforms to reconstruct the evolution of the FIS drainage from both direct and indirect evidence. The oldest MSGLs observed, located on surface N8, suggest that the inception of fast flow within the FIS took place at a tentative age of ∼1.6–1.9 Ma. During that period, ice flowed to the southwest from the Vestfjorden area, reaching the palaeo-shelf break, which was beneath modern Traenabanken (Fig. 8). By the beginning of deposition of Sequence A, another fast, westward flowing sector of the FIS developed in the Haltenbanken-Suladjupet area and southwest to north-northwest flow-switching took place on Traenabanken (Fig. 8). Relatively small slope depocentres and an absence of palaeo-troughs in the shelves of the younger N units provide no conclusive evidence for significant focused erosion related to fast ice-flow. Large depressions shown by 2D seismic data within the preserved inner shelf of surface A0 suggest that the inception of the first erosive ice stream may have been as early as ∼1.5 Ma; the lateral margins of several eroded troughs are shown in Fig. 8.

**Fig. 9:**
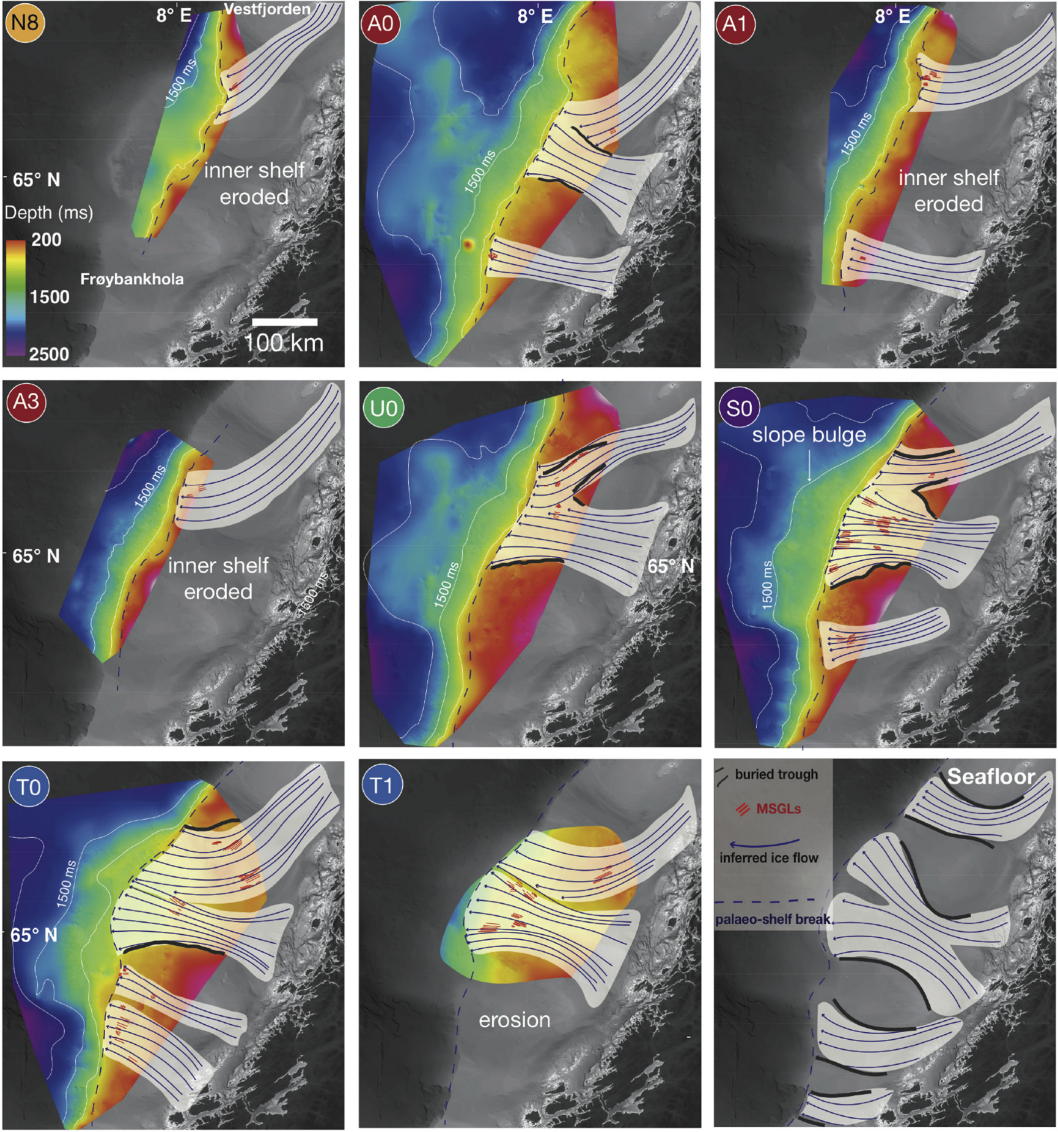
**Evolution of ice streams in the Fennoscandian Ice Sheet on the mid-Norwegian shelf**. Coloured surfaces represent structure maps of interpreted seismic horizons. Red lines indicate MSGLs providing direct evidence of fast flow found within the palaeo-surfaces of the Naust Formation. Dark blue lines show the inferred extrapolated patterns of fast flow based on the MSGLs and more general topographic setting. White lines show slope topographic contours for 1000, 1500 and 2000 ms (two-way travel-time). Thick black lines show interpreted edges of palaeo-troughs. (For interpretation of the references to colour in this figure legend, the reader is referred to the web version of this article.)

The basal surface of Sequence U (tentative age ∼800 ka) shows at least two major converging cross-shelf troughs occupied by sets of MSGLs (Fig. 4e). Intricate cross-cutting and overprinting relationships between streamlined and transverse landforms (Fig. 4e) suggest complex ice flow during individual glacial cycles since at least the middle Quaternary (Fig. 4d and e). In the Skjoldryggen area, a major slope depocentre containing abundant debris flows, known to be the building blocks of late Weichselian trough-mouth fans (*e*.*g*. Laberg and Vorren, 1995) suggests that the FIS palaeo-ice streams reached full-glacial conditions during Naust U time. However, making direct palaeo-drainage reconstructions during the few glaciations following the deposition of the ∼800 ka old surface U0 is problematic since the palaeo-shelves within the younger surfaces U1–U5 were completely removed by major Elsterian (∼400–320 ka) and Saalian (∼300–140 ka) ice-sheet expansions. The development of extremely erosive Elsterian and Saalian ice flow is illustrated by complex regional erosion surfaces and the widening of the palaeo-troughs beneath the modern Sklinnadjupet and Traenadjupet areas (Fig. 8). To the south, in the Haltenbanken-Suladjupet area, divergent streamlined landforms provide evidence for ice flowing southwestwards across modern Haltenbanken to the palaeo-shelf break (Fig. 8, Fig. 9). During the penultimate, Saalian glaciation, this ice stream experienced southwest-northwest flow switching (Fig. 8, Fig. 9). The latest episode of regional flow-switching occurred in Traenadjupet area during the last, Weichselian glaciation (∼10–115 ka) (Dowdeswell et al., 2006).

## 7 Discussion and implications

### 7.1 Changing ice-sheet flow during the Quaternary

Buried subglacial landforms and iceberg ploughmarks found within multiple Naust units provide evidence for the palaeo-glaciological reconstruction of marine-terminating sections of the FIS from the earliest Quaternary (Fig. 7, Fig. 9). Buried ploughmarks are found on almost every interpreted surface (Fig. 4), suggesting that drifting icebergs were present on the continental shelf through at least a part of most cold periods of the Quaternary. The oldest iceberg ploughmarks are found close to the basal Naust palaeo-surface (Fig. 4a), which is roughly 0.8 Ma older than the oldest ploughmarks previously reported from the mid-Norwegian shelf (Dowdeswell et al., 2007, Ottesen et al., 2009). This observation suggests the presence of a calving FIS margin reaching the Norwegian coast since at least the beginning of the Quaternary, with implications for potentially increased iceberg discharge and freshwater flux into the North Atlantic Ocean ∼0.8 Ma earlier in the Quaternary than was previously thought.

Although ploughmarks demonstrate that the FIS has reached the western coast of Norway to produce icebergs since the beginning of the Quaternary, there are no subglacial landforms diagnostic of fast ice flow found during the early Quaternary (∼2.7–1.5 Ma). MSGLs found within the upper N and lower A units suggest the presence of at least two episodes where fast-flowing ice streams were present on the shelf (Fig. 4), tentatively ∼1.6–1.4 Ma. The initiation of fast flow requires a significant mass flux from the adjacent interior drainage basin (*e*.*g*., Bentley, 1987), suggesting that, by that time, the FIS was large enough to supply the necessary amount of ice to its marine western margin.

Since the middle Quaternary (∼0.8 Ma), at least four major, erosive, fast-flowing ice streams formed within the FIS, excavating troughs across the continental shelf (Fig. 4, Fig. 9). Evidence from diagnostic MSGLs demonstrates that two of these ice-stream episodes experienced major flow-switching events since the Elsterian glaciation (Fig. 8). On glacial-interglacial timescales (i.e., 50–100 ka), these spatial and temporal variations in palaeo-drainage patterns show a dynamic FIS, with complex and evolving ice flow.

### 7.2 Development of ice-sheet erosional intensity

**Fig. 10:**
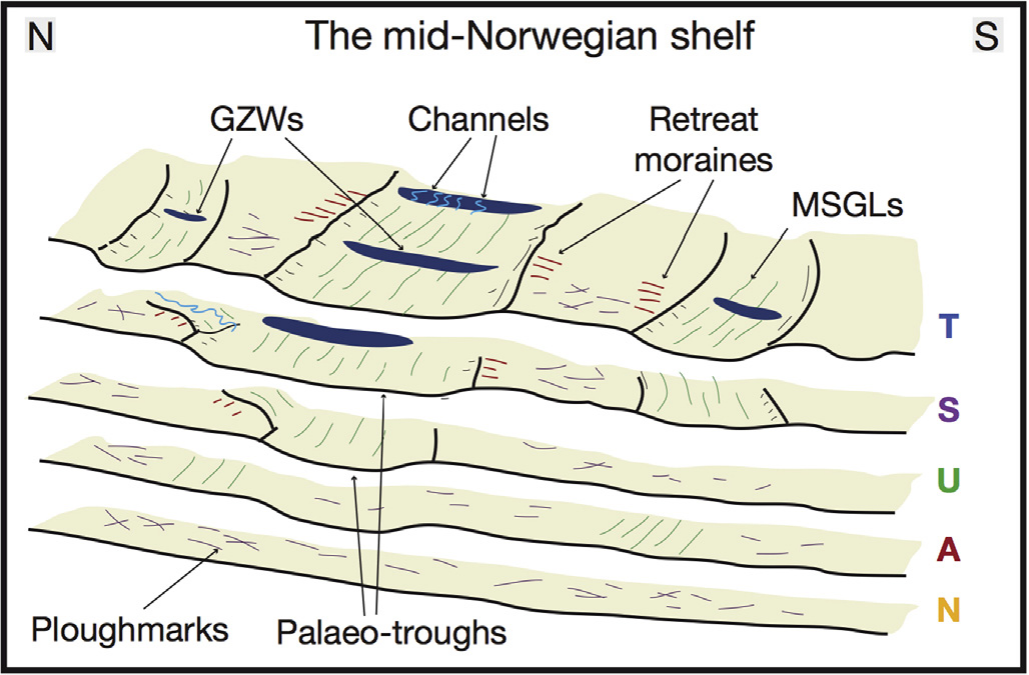
Idealised sketch of the Quaternary development of the outer- and mid-shelf sections of the mid-Norwegian shelf inferred from the summary of landform occurrence provided in Table 1.

Detailed thickness maps produced for glacially derived sediments from multiple Naust units demonstrate a significant increase in slope sediment flux beyond the buried cross-shelf troughs since ∼0.8 Ma (Fig. 7). This evidence shows that, throughout their development, the FIS ice streams gradually strengthened their erosional intensity (Fig. 10). The tentative timing of onset of this enhanced sediment delivery coincides with the mid-Pleistocene Transition (∼1.2–0.7 Ma, Clark et al., 2006) and intensification of the Northern Hemisphere glaciations, suggesting that orbital forcing may have played a role in the increased erosive capacity of the FIS's large ice streams (Fig. 2c).

Another criterion demonstrating the evolution of the FIS's erosive capacity is the variable degree of preservation of palaeo-surfaces. Accordingly, outer shelves of Sequences N and A, that were formed during 41 ka glacial cycles, are largely preserved (Table 1), whereas palaeo-shelf surfaces of younger Sequence U are completely removed by erosion that occurred when longer 100 ka cycles were dominant (Fig. 2b). The most prominent erosion, associated widening of cross-shelf troughs and deposition of thick GZWs, occurred during the last three glaciations, when the periods of glacial maxima were colder and longer, as shown by the global oxygen-isotope record (Fig. 2c) (Lisiecki and Raymo, 2005).

Variable preservation of palaeo-surfaces in the mid-Norwegian shelf could also be impacted by other factors than changing erosional intensity on its own. Erodibility of the shelf sediments represents another factor that can influence the preservation of palaeo-shelves, but this parameter is often hard to estimate as many of the palaeo-shelves have been removed and reworked by successive glaciations. Shelf subsidence, resulting from loading by sediment delivery from successive full-glacials, would create accommodation space and facilitate the preservation of palaeo-surfaces, as demonstrated by units N and A, where most of the gently dipping outer shelves have survived subsequent ice readvances (Dowdeswell et al., 2007). Lastly, deposition of thick sedimentary grounding-zone wedges above a palaeo-surface could also serve as a ‘shield’ protecting buried surfaces from being eroded by the next phase of ice-sheet growth, as in the case of the almost entirely preserved palaeo-shelf surface T0, which is covered by the series of thick, partly eroded massive units deposited within the Sequence T.

Whereas climate probably provides first-order control on the regional ice-sheet mass balance and, hence, initiation and intensification of fast flow within the FIS, the onset of fast flow in a particular area and flow-switching from one location to another depends on more local variables, primarily subglacial topography, geology and hydrology (*e*.*g*., Blankenship et al., 1987, Bell et al., 1998, Winsborrow et al., 2010). The Vestfjorden ice stream (Fig. 1, Fig. 8), for example, was confined to a deep trough, and major change in the location of its terminus was probably influenced by the Saalian deposition of thick GZWs that substantially altered the shelf topography, providing an obstruction to the pathway of the ice stream during the following (last) glacial cycle, when Traenadjupet was cut (Dowdeswell et al., 2006). Since deposition of GZWs requires still-stands in the terminus of an ice stream (*e*.*g*., Dowdeswell and Fugelli, 2012), the style of retreat of the FIS and the resulted sedimentation may have exerted considerable impact on the topography of the shelf, thus influencing the flow-switching observed in both Traenadjupet and Haltenbanken areas (Dowdeswell et al., 2006). In contrast, until the last glaciation, the Frøybankhola ice stream was not associated with a clear, prominent trough similar in dimensions to its northern neighbours. Whereas the terminus of the ice stream remained in roughly the same location, the MSGLs show an almost 90° change in flow direction towards the inner shelf (Fig. 8). These observations suggest different timing and potentially variable controls on flow-switching, including major reorganisations of interior ice-sheet drainage basins and changes in the subglacial hydrological system.

### 7.3 Subglacial hydrology and ice-sheet thermal regime

Multiple surfaces extracted from the ∼40,000 km^2^ 3D seismic record of the mid-Norwegian shelf document little conclusive evidence for the presence of an erosive subglacial hydrological system detectable by the relatively high-resolution seismic data (Table 1), despite grounded ice reaching the palaeo-shelf break several times since the early Quaternary. This observation is in stark contrast with neighbouring regions of the North Sea, located ∼500 km to the south, where multiple generations of prominent, deep tunnel valleys cut by meltwater have been mapped within the Late Quaternary sediments (*e*.*g*., Stewart and Lonergan, 2011) and the Barents Sea, where a temperate Pliocene-middle Pleistocene ice sheet has been inferred from the channels preserved within the buried palaeo-surfaces (Laberg et al., 2010). The observed lack of extensive subglacial meltwater channels, complemented by evidence of channel-fan complexes located within buried GZWs (Fig. 5), suggests a largely distributed, low-volume meltwater system that may have drained the FIS through permeable subglacial till without leaving much erosional evidence (*e*.*g*., Blankenship et al., 1987, Tulaczyk et al., 2001, Dowdeswell et al., 2004). The meltwater could originate either from the surface of the ice sheet during deglaciation or subglacially as a result of geothermal and strain heating, suggesting an intermediate to cold glacimarine setting during the formation of these features (Dowdeswell et al., 2016). However, we cannot rule out the possibility that an abundant channelized meltwater system either existed in a form of small, sub-seismic scale channels (i.e., <10 m wide) or was present on the eroded shallower inner shelves of the study area, as previously reported from some areas of the continental shelf of Antarctica (*e*.*g*., Wellner et al., 2001, Larter et al., 2009).

### 7.4 Wider implications

The detailed, margin-wide reconstruction presented here (Fig. 7, Fig. 9) elucidates the key aspects of the evolution of the western FIS on the mid-Norwegian margin that are essential for modelling ice-sheet – climate feedbacks (*e*.*g*., Bell, 2008, Stokes et al., 2015). In addition, the positions and volumes of local depocentres (Fig. 7) provide useful constraints for modelling topographic evolution and isostatic adjustments associated with glacial deposition and erosion at long timescales (*e*.*g*., Steer et al., 2012, Hall et al., 2013). Although the early evolution of the Naust Formation is chronologically poorly constrained, the exceptional spatial data coverage and level of detail of ice-sheet reconstructions presented here make the mid-Norwegian continental margin a key location for future ocean drilling programs that aim to investigate the dynamics of past ice sheets on glacial-interglacial timescales.

The fragmentary preservation of buried surfaces and diagnostic landforms, summarised in Table 1, also illustrates limitations on ice-sheet reconstructions even in a region with exceptionally extensive 3D data coverage. For instance, only ∼40 individual MSGLs confined within small sectors of up to ∼100 km^2^ were found on the outer shelves of Sequences N and A, making it problematic to infer changes in the dimensions of palaeo-ice streams in the early Quaternary. In the Haltenbanken area, no evidence of fast flow was found buried deeper than the base of Sequence U (Fig. 9), although adjacent slope depocentres show that focused sediment delivery and MSGLs are present in both Sequences A and S (Fig. 7). Moreover, largely eroded inner shelves preclude the investigation of inner ice-sheet drainage configuration and the potential presence of meltwater channels in shallower parts of the margin. Thus, the absence of landforms diagnostic of particular subglacial processes does not necessarily imply that those processes were inactive. We emphasise that complex ice-sheet dynamics, with high spatial and temporal variability, together with inherent limitations caused by glacial erosion, should be taken into account when reconstructing the dynamics of an ice sheet from datasets with limited spatial coverage.

## 8 Conclusions

Extensive 3D and 2D high-resolution seismic datasets covering the vast (∼150,000 km^2^) mid-Norwegian continental margin provide the largest archive of recorded past ice-sheet activity available from a high-latitude region. Multiple surfaces interpreted from these data show various buried landforms diagnostic of past glacial processes, illustrating the evolution of the FIS on glacial-interglacial timescales. These features range in scale and include buried cross-shelf troughs, mega-scale glacial lineations, grounding-zone wedges, recessional push moraines, iceberg ploughmarks, meltwater channels and a hill-hole pair (Fig. 3).

Large numbers of irregular iceberg ploughmarks found on the palaeo-seafloors within the Quaternary Naust Formation show that a calving margin of the FIS was present on the Norwegian coast since the earliest Quaternary, ∼0.8 million years than previously suggested. Landforms indicative of fast ice flow show the gradual development of fast-flowing ice streams since ∼1.5 Ma and erosional intensification from ∼0.8 Ma marked by incision of the first large cross-shelf trough (Fig. 9). Depositional evolution of the mid-Norwegian shelf and slope show a significant increase in slope sediment flux beyond the buried cross-shelf troughs since ∼0.8 Ma (Fig. 7), suggesting that throughout their development the FIS ice streams gradually strengthened their erosional intensity (Table 1). Subglacial landforms reveal a complex and dynamic ice sheet, with converging palaeo-ice streams and several flow-switching events that may reflect major changes in topography and basal thermal regime. Lack of large subglacial meltwater channels suggests a largely distributed drainage system beneath the marine-terminating part of the FIS.

This palaeo-environmental examination of the FIS provides a useful framework for ice-sheet modelling and planning of the future scientific drilling sites. Fragmentary preservation of buried surfaces and variability of ice-sheet dynamics revealed by this detailed, regional study emphasise that care should be taken when reconstructing glacial history of large ice sheets from spatially limited datasets.

## Figures and Tables

**Table 1:** **Summary of the geomorphological features and preserved buried surfaces found in the mid-Norwegian margin.** T- cross-shelf troughs; MSGLs – mega-scale glacial lineations; GZWs – grounding-zone wedges. The variable number of preserved palaeo-surfaces in different sectors of the mid-Norwegian margin shows mostly eroded inner shelves and largely preserved slopes. The approximate number of interpreted features is indicated for each type of landform. For iceberg ploughmarks, average length is indicated in kilometres in brackets.

Sequence	Preserved surfaces number/name			Buried landforms					
	Inner shelf	Outer shelf	Slope	T	MSGLs	GZWs	Retreat moraines	Channels	Ploughmarks
T	2 (T0, T1)	2 (T0, T1)	2 (T0, T1)	5	>250	4	∼35	∼10	>3000 (3.0)
S	1 (S0)	1 (S0)	3 (S0–S2)	3	>250	1	∼50	1	>700 (2.7)
U	1 (U0)	1 (U0)	6 (U0–U5)	2	>100	0	∼25	0	>600 (3.2)
A	1 (A0)	4 (A0–A3)	5 (A0–A4)	1	∼30	0	0	0	>1600 (3.1)
N	1 (N0)	9 (N0–N8)	10 (N0–N9)	0	∼10	0	0	0	>600 (2.4)
